# Heroes of peer review: Robert Lowe

**DOI:** 10.1186/s13059-017-1208-0

**Published:** 2017-04-19

**Authors:** Robert Lowe

**Affiliations:** The Blizard Institute, Barts, and The London School of Medicine and Dentistry, Queen Mary University of London, London, E1 2AT UK

## Abstract

In the second of our series of articles celebrating peer reviewers, we talk to Robert Lowe, who is a Lecturer in computational biology at Queen Mary University of London, UK, and a valued reviewer for *Genome Biology*.

## Please tell us about your current research interests

I use computational tools to better understand epigenetic processes. The starting point for this work has been the identification of biological processes that involve clear and robust differences in epigenetic marks.

In 2015 [1], we identified senescence-associated differentially methylated positions (senDMPs) in human mammary epithelial cells. Interestingly, we found that these senDMPs could be reversed by transfecting cultures with small-interfering RNAs (siRNAs) targeting p16. This therefore provides us with an opportunity to investigate the dynamics of DNA methylation through this process. In particular, we are interested in how regions of CpGs become methylated and unmethylated. Previously, Landan and colleagues [2] have shown that development of differential methylation occurs through a stochastic process during in vitro evolution of immortalized fibroblasts, and Landau et al. [3] have shown similar stochastically disordered methylation in malignant cells of chronic lymphocytic leukaemia, which was associated with adverse clinical outcome. The question therefore remains—do all regions of differential methylation occur through stochastic processes?

We are also interested in the interplay between epigenetic modifications, such as histone modification and DNA methylation, as well as between epigenetic modifications and genetics. We recently identified [4] variability in DNA methylation in response to early-life nutrition at ribosomal DNA (rDNA), which is restricted to rDNA copies associated with a specific genetic variant.

Our aim is to build computational models of these interesting biological processes that we hope will provide a deeper understanding and lead to predictions that we can then test.

## What are your predictions for your field over the next 5 years?

There are a number of key challenges in epigenetic research that will hopefully start to be addressed in the next 5 years.

We need to understand the contribution that epigenetic variation actually plays in the various biological processes that we see correlations with, such as cancer, ageing and cellular differentiation. It is possible that a number of these epigenetic marks are present owing to genetic variation or are recruited after transcriptional changes occur and hence do not result in any functional consequence. With the use of CRISPER-Cas9 technologies and the possibility of directly changing DNA methylation, it should be possible to interrogate how important a role epigenetics actually plays.

Additionally, we need to start to understand the important context of where these epigenetic variations occur. The function of the same epigenetic marks can be different at different locations within the genome, and therefore understanding what the defining features of these locations are is important. For example, it is becoming ever more clear that the three-dimensional structure of the genome allows for the interpretation of seemingly long-range interactions by means of the looping of the genome.

Finally, as it becomes increasingly cost effective to profile many different biological processes, including the transcriptome, the metabolome and the proteome, it will become necessary to incorporate these datasets together. This could provide a unique opportunity to begin to understand the potential effects and causes of the variation in the epigenetic marks.

## What motivates you to provide peer review for journals?

It is vitally important that the scientific community upholds a form of quality control in which work can only be published with the appropriate data from which conclusions are drawn. While peer review is clearly not perfect, I believe it is an important and fundamental part of science, and therefore I see it as an important part of my job. An additional motivator is remembering the many hours that have been volunteered by my scientific colleagues in providing peer review for my own papers.



## What changes, if any, would you make to the current system of peer review?

The main change I would make to peer review is to make the process double-blind and not allow the reviewers to know the identity of the authors. I think that the process should be based purely on the work presented, and I think that reviewers can often be persuaded to accept or reject an article based on who is submitting it.

I would also be interested in the ability to submit questions to the authors in a less formal way. For example, it should be possible during the review process to contact the authors and ask for clarification on small points that can often be crucial for the understanding of the paper. This would also provide the opportunity for a detailed discussion on a much quicker timescale than is currently possible through the review and resubmit process. The reviewer could then, based on this detailed discussion, submit a formalised question in which the authors would be able to respond effectively as they would understand fully the aim of the question.
